# Perceptions of managerial competencies, style, and characteristics among professionals in nursing

**DOI:** 10.3325/cmj.2011.52.198

**Published:** 2011-04

**Authors:** Mateja Lorber, Brigita Skela Savič

**Affiliations:** 1 Faculty of Health Sciences, University of Maribor, Maribor, Slovenia; 2 College of Nursing Jesenice, Faculty of Management Koper, University of Primorska, Jesenice, Slovenia

## Abstract

**Aim:**

To compare nursing leaders’ and employees’ perception of leaders’ leadership style, personality characteristics, and managerial competencies and to determine the associations between these factors.

**Methods:**

The study included 4 out of 5 Slovenian major hospitals selected from the hospital list; 1 hospital refused to participate. The employees of these hospitals represent 30% of all employees in nursing in Slovenian hospitals and the 509 employees included in the study represent 6%. One structured survey questionnaire was administered to the leaders and the other to employees, both consisting of 134 statements evaluated on a 5-point Likert-type scale. The relationship between demographic data, leadership style, leaders’ personality characteristics, and leaders’ training and managerial competencies was analyzed by correlation and multivariate regression analysis. The study took place in April 2009.

**Results:**

Leaders and employees significantly differently evaluated 13 out of 14 managerial competencies of the leaders, where leaders rated themselves higher for vision and goals, communication, conflict resolution-agreement, compromise, adjustment, motivation, interpersonal relationships, problem solving, delegation, teamwork, decision making, emotional intelligence, and human resources development. Employees rated the leaders higher for managing changes and conflict resolution-dominance and avoidance. Multivariate regression analysis showed that managerial competencies were explained by leadership style, leaders’ training, leaders’ characteristics, and type of employment in 86.1% of cases.

**Conclusion:**

Leaders in nursing too frequently used inappropriate leadership style. Forming a unique model for all health care institutions in the country would facilitate the evaluation of competencies and constant monitoring of leaders’ work results.

Competencies are a relatively new name for knowledge, skills, abilities, and virtues, which have been known and used for a long time. Boyatzis (1) was the first to use the term competency, considering it a combination of motivation, skills, self-image, and social roles. Competencies comprise knowledge, cognitive and practical skills, values, attitudes, abilities, and behaviors (2-4) that are demonstrated as successful and efficient performance at the workplace. New (5) defines managerial competencies as a way in which an individual cooperates with other individuals, while Hudak et al (6) define it as the skills, knowledge, and abilities required for achieving quality.

The skills and knowledge sets required for nurse leaders are well-known (2,7,8). The American Organization of Nurse Executives (9) maintains that leaders must be competent in communication and relationship-building, knowledge of the health care environment, professionalism, and business skills. Managerial competencies have been investigated by different approaches. Heller et al (10) identified 6 essential managerial competencies for nurse leaders: interpersonal communication, organizational navigation, crisis management, time management, and adaption, whereas Kagan et al (11) identified human relations, communications, teamwork, problem solving, credibility, openness, and staff development. Cadmus (12) identified self-management, communication, leadership style, introduction of innovations and changes, and quality. In 2007, Leadership Alliance defined 5 competency domains common to all practicing health care leaders: communication and relationship, professionalism, leadership, knowledge of the health care system, and business skills (13). In the reports published in the same year, Jennings et al (3) identified 10 managerial competencies for nursing leaders: personal qualities, interpersonal skills, thinking skills, setting the vision, communicating, initiating change, developing people, health care knowledge, and management and business skills, while Schira (14) listed communication, openness, motivation, vision, development, passion, risk taking, environmental control, and giving rewards.

In Slovenia, workplace description rarely includes the definition of nursing managerial competencies for different qualification levels and they vary considerably among different hospitals. To be able to better define managerial competencies, this study compared the perception of nursing leaders’ leadership style, personality characteristics, and managerial competencies between nurse leaders and employees and determined the associations between these factors.

## Methods

### Sample and study design

The study took place in April 2009 in 4 major Slovenian hospitals – University Clinical Center Maribor, General Hospital Celje, General Hospital Slovenj Gradec, and General Hospital Murska Sobota. Five major Slovenian hospitals had been selected from the hospital list, but 1 refused to participate. Employees in the 4 participating hospitals represented 30% of employees in nursing in all Slovenian hospitals. The questionnaires were distributed in the morning shift, by authors in one hospital and by research coordinators in other 3 hospitals. There were 750 questionnaires distributed, which amounts to 26.8% of 2802 employees in nursing in Slovenian hospitals that participated in the study and 8% of 9404 employees in nursing in all Slovenian hospitals. Hundred and ten questionnaires were sent to middle- and unit-level nurse leaders and 640 to other nursing employees. Nurse leaders were not selected randomly; the questionnaires were sent only to those with a job relevant for the research, which means that purposive sampling was used. Maximum time for filling out the questionnaire was 14 days. Questionnaires were collected in specially designed boxes to ensure anonymity. Five hundred and nine questionnaires were correctly and completely filled out and the response rate was 68%. This sample represented 6% of all employees in nursing in Slovenian hospitals. The 4 hospitals had provided a written permission for research.

### Instruments

Two survey questionnaires with 134 closed-type questions each were used, one for leaders and one for employees in nursing (web extra material)(web extra material 1). The questionnaires were prepared based on the literature on modern leadership and managerial competencies of nursing leaders (3,10,11,14-18) and in cooperation with the O.K. Consulting (company for education and research of employees in all areas), and had been tested in a pilot study (10 leaders and 30 employees). Leaders self-assessed their leadership style, managerial competencies, and characteristics on a 5-point Likert scale ranging from 1 (strongly disagree) to 5 (strongly agree). Employees assessed the leadership style, managerial competencies, and characteristics of their immediate superior on a 5-point Likert scale ranging from 1 (strongly disagree) to 5 (strongly agree). The first part of the questionnaire inquired about demographic data: sex, age, institution, years of employment, years of employment in a leading position, and the level of education. The second part contained statements on 14 managerial competences (0.691-0.908): social power (Cronbach α 0.779), vision and goals (0.855), communication (0.789), motivation (0.818), conflict resolution (0.722), interpersonal relations (0803), problem solving (0.908), team work (0.899), decision making (0.774), delegating (0.691), managing changes (0.701), emotional intelligence (0.731), human resources development (0.798), and quality (0.735).

### Statistical analysis

We used *t* test and correlation analysis, as well as multivariate regression analysis, to determine the association between factors. The program used was SPSS, version 16.0 (SPSS Inc., Chicago, Illinois, USA).

## Results

Leaders’ median age was 43.5 years (33-59 years) and employees’ 38 years (21-60 years). The sample included 11 men and 496 women. The proportion of men among leaders was 1% and among employees 2.4%, which corresponds to the proportion of men in nursing in Slovenia (19) and in nursing in general (20,21). Our participants became leaders at the median age of 38 years and spent an average of 10.1 years in the leading position (10% had been in the leading position for a year or less and 6.3% over 25 years).

Table 1 shows that leaders in nursing mostly used task- and relationship-oriented leadership style (mean ± standard deviation, 4.14 ± 0.4), followed by task-oriented leadership style (4.00 ± 0.4) and relationship-oriented leadership style (4.00 ± 0.4). The fewest leaders used a leadership style characterized by low orientation to task and relationship (2.86 ± 0.5) (22,23).

**Table 1:** Perceptions of leaders’ leadership style by Slovenian leaders (self-assessment) and employees in nursing

Leadership style	Perceptions (points) by:		*t*^†^	*P*
	leaders	employees		
	mean ± standard deviation	mean ± standard deviation		
Relationship- and task-oriented	4.14 ± 0.4	3.79 ± 0.6	6.294	<0.001
Characterized by low orientation to task and to relationship	2.86 ± 0.5	3.13 ± 0.5	-5.159	<0.001
Relationship-oriented	4.00 ± 0.4	3.79 ± 0.7	3.391	<0.001
Task-oriented	4.00 ± 0.4	3.56 ± 0.6	9.034	<0.001

*Mean (on a scale from 1 to 5), 5 questions for each leadership style.

†*t* test.

There were significant differences between leaders’ and employees’ perceptions of all 4 leadership styles. Leaders thought that they more often used task- and relationship-oriented leadership style (*t* = 6.170; *P* < 0.001), task-oriented leadership style (*t* = 9.061; *P* < 0.001), and relationship-oriented leadership style (*t* = 3.699; *P* < 0.001). However, employees believed that leaders often used a leadership style characterized by low orientation to task and relationship (*t* = -5,159; *P* < 0.001). Table 2 shows the results of computed value of *t* test for managerial competencies. Leaders and employees significantly differed in the perception of 13 out of 14 managerial competencies. Leaders thought that they more often used managerial competencies such as vision and goals, communication, conflict resolution (agreement, compromise, and adjustment), motivation, interpersonal relations, problem solving, delegating, team work, decision making, emotional intelligence, human resources development, and quality, compared with the employees’ evaluation (Table 2). Employees assessed that leaders avoided conflicts (*t* = -5.153; *P* < 0.001) and that they used dominance to solve them (*t* = -4.985; *P* < 0.001). Employees assessed leaders’ characteristics and leaders self-assessed their own personality characteristics. They differed in the perceptions of the following variables (Table 3): decisiveness, initiative, innovation, ambition, persistence, communication, self-confidence, adaptability, activity at work, tact, thoughtfulness, honesty, sociability, reliability, objectivity, cooperation, teamwork, organizational skills, responsibility, and emotional intelligence. Leaders evaluated their characteristics higher than employees, except ambition, self-confidence, and sociability. Leaders and employees ranked the 10 most important characteristics of leaders. The most important characteristic was honesty, followed by organizational skills, teamwork, decisiveness, reliability, objectivity, responsibility, self-confidence, communication skills, and ambition (Table 3). These 10 characteristics were used as selected characteristics of ‘good leaders.’

**Table 2:** Perceptions of leaders’ managerial competencies by Slovenian leaders (self-assessment) and employees in nursing

Managerial competencies	Perceptions (points) by:		*t*^†^	*P*
	leaders	employees		
	mean ± standard deviation*	mean ± standard deviation		
Social power	4.19 ± 0.4	4.09 ± 0.6	1.828	0.069
Vision and goals	4.19 ± 0.4	3.89 ± 0.6	5.808	<0.001
Communication	3.97 ± 0.3	3.74 ± 0.5	5.357	<0.001
Conflict resolution – agreement	4.50 ± 0.6	3.92 ± 0.9	7.595	<0.001
Conflict resolution – compromise	4.08 ± 0.6	3.85 ± 0.9	3.030	0.003
Conflict resolution – dominance	2.82 ± 1.3	3.53 ± 1.0	-4.985	<0.001
Conflict resolution – adjustment	4.05 ± 0.8	3.77 ± 0.9	3.075	0.002
Conflict resolution – avoidance	1.78 ± 1.0	2.40 ± 1.2	-5.153	<0.001
Motivation	4.17 ± 0.3	3.49 ± 0.5	16.129	<0.001
Interpersonal relations	4.12 ± 0.4	3.75 ± 0.6	7.679	<0.001
Problem solving	4.26 ± 0.4	3.94 ± 0.7	6.036	<0.001
Delegating	3.90 ± 0.4	3.73 ± 0.6	3.353	<0.001
Teamwork	4.54 ± 0.4	4.01 ± 0.7	10.197	<0.001
Decision making	3.57 ± 0.4	3.46 ± 0.5	2.516	0.013
Managing changes	3.79 ± 0.3	3.88 ± 0.5	-2.598	0.010
Emotional intelligence	4.16 ± 0.5	3.92 ± 0.6	3.903	<0.001
Human resource development	4.12 ± 0.4	3.75 ± 0.6	7.198	<0.001
Quality	4.27 ± 0.4	3.90 ± 0.6	7.838	<0.001

*Mean (on a scale from 1 to 5).

†*t* test.

**Table 3:** Perceptions of leaders’ personality characteristics by Slovenian leaders (self-assessment) and employees in nursing

Personality characteristics	Perceptions (points) by:		*t^†^*	*P*	Rank
	leaders	employees			
	mean ± standard deviation*	mean ± standard deviation			
Decisiveness	4.49 ± 0.6	4.27 ± 0.9	2.898	0.002	4
Initiative	4.39 ± 0.7	4.07 ± 0.9	3.442	<0.001	
Innovation	4.31 ± 0.7	4.05 ± 0.9	2.540	0.007	
Ambition	3.96 ± 0.9	4.25 ± 0.9	-2.993	0.005	10
Persistence	4.53 ± 0.6	4.38 ± 0.8	3.083	<0.001	
Communication	4.59 ± 0.6	4.38 ± 0.9	2.705	0.005	9
Self-confidence	4.24 ± 0.7	4.35 ± 0.8	-1.218	0.295	8
Adaptability	4.43 ± 0.6	4.08 ± 1.0	3.343	<0.001	
Activity at work	4.76 ± 0.5	4.26 ± 0.9	7.568	<0.001	
Tact	4.31 ± 0.7	4.00 ± 0.9	3.096	0.002	
Thoughtfulness	4.46 ± 0.6	4.11 ± 0.9	4.484	<0.001	
Honesty	4.86 ± 0.5	4.27 ± 0.9	9.586	<0.001	1
Sociability	4.33 ± 0.7	4.38 ± 0.8	-0.499	0.727	
Reliability	4.79 ± 0.6	4.39 ± 0.8	7.068	<0.001	5
Objectivity	4.46 ± 0.4	4.00 ± 0.9	6.188	<0.001	6
Cooperation	4.38 ± 0.6	4.08 ± 0.9	3.223	0.002	
Teamwork	4.70 ± 0.7	4.24 ± 0.9	6.958	<0.001	3
Organization skills	4.57 ± 0.5	4.16 ± 0.9	5.940	<0.001	2
Responsibility	4.76 ± 0.5	4.40 ± 0.8	5.674	<0.001	7
Emotional intelligence	4.34 ± 0.8	4.10 ± 0.9	2.319	0.031	

*Mean (on a scale from 1 to 5).

†*t* test. Only the first 10 ranks are indicated.

### Regression analysis of managerial competencies

Simpler variance models revealed an important factor in all managerial competencies – position (leaders had a higher expected score than employees). Multiple regression analyses included the following independent factors: demographic data (age, years of employment, level of education), leaders’ training, leadership style, and selected characteristics of good leaders. Table 4 shows the results of the regression analysis of managerial competencies. The studied managerial competencies were related to the level of education (β = 0.042; *P* = 0.033; SE = 0.010), type of employment (β = 0.156; *P* < 0.001; SE = 0.027), leaders’ training (β = 0.330; *P* < 0.001; SE = 0.020), leadership style (β = 0.536; *P* < 0.001; SE = 0.029), and leaders’ characteristics (β = 0.184; *P* < 0.001; SE = 0.018). These factors accounted for 86.1% of the variability of the studied managerial competencies. Among these factors, leadership style had the most significant impact on managerial competencies (β = 0.536).

**Table 4:** Results of regression analysis for perception of leaders’ managerial competencies by Slovenian leaders and employees in nursing

Variables*	b	Standard error		β	*P*
Age (year)	0.001		0.002	0.474	0.636	
Level of education	0.021		0.010	0.042	0.033	
Type of employment	-0.197		0.027	-0.156	<0.001	
Leaders’ training	0.283		0.020	0.330	<0.001	
Leadership style	0.630		0.029	0.536	<0.001	
Leaders’ characteristics	0.148		0.018	0.184	<0.001	

*R^2^ = 0.861.

## Discussion

The study showed that leaders perceived their managerial competencies better than employees did. Also, self-confidence was found to be among 10 most important characteristics of successful leaders. Shipper et al (15) and Rahim et al (15) also found that self-confidence was connected with the leadership success. Similar was also found for good organizational and communication skills. However, we showed that nursing leaders were not good listeners, they did not give the employee a sense of equality, and they did not adjust their communication to different people and situations, and sometimes even imposed their opinion on their coworkers.

In our study, leaders’ choice of conflict-solving strategy was influenced by leadership style and age, similar to other studies that showed that it was influenced by leadership style and length of tenure (24).

Nurse leaders in Slovenian hospitals were not adequately trained to solve conflicts, since they often solved them by adjustment and agreement and did not use reshaping methods, which would partly or entirely change the conflict situation (25).

Improved managerial competencies were found to decrease conflicts and improve teamwork (26). Although nursing leaders in our study did not have good communication skills and showed a lack of concern for good interpersonal relations, both they and the employees highly valued good interpersonal relations. Other studies have also shown a lack of cooperation and communication between employees in nursing (27).

We showed that praise influenced motivation and satisfaction of leaders and employees. It is important that leaders are aware that motivated employees are a source of competitive edge. They need motivating acknowledgment, praise, encouragement, feedback, opportunities to take responsibility, consistency, and sincerity from their leaders and coworkers, and job security (27). Nurses who are motivated and satisfied with their jobs will stay at the workplace and contribute to the success of the organization.

We found that leaders often delegated more demanding tasks to their coworkers than they were qualified to perform. Similar results were also obtained by other authors (28), who found that communication between all members is crucial for the functioning of a nursing team. Both physicians and nurses in Slovenia estimated their level of personal involvement and involvement in work teams as insufficient (29), while discrepancy in perception of team work between nurses and physicians caused suboptimal conflict resolution and inadequate interpersonal communication (30).

This study also showed that nursing leaders did not involve their employees in the decision-making process and in the management of change, although this has been demonstrated to be important (25). Moreover, nursing leaders in our study still used different kinds of pressure, their personal power, and the power of management hierarchy in the decision-making process. They were aware that it would be necessary to introduce more changes to improve the quality of nursing, but they preferred to avoid it. Also, they did not present the changes well to the employees and poorly anticipated obstacles in managing changes. As already mentioned, teamwork is the key factor for a successful introduction of change in Slovenian hospitals (31) and has been found to make nursing care increasingly responsive and proactive (32).

Leaders must be sensitive to the feelings of other people and must be able to react calmly, with ease, and in a controlled manner in different stressful situations. Leaders in our study inadequately recognized emotional states of their coworkers, which means that they had poorly developed empathy. They also did not know how to stay rational in difficult and stressful situations, and did not express their anger in an appropriate way. Empathy, as an element of emotional intelligence, is a sign of managerial advantage and success (16,33,34). The nurses whose superiors have a pronounced emotional intelligence have good mental health and are also more likely to provide quality nursing care of their patients (16).

Improving work processes in nursing leads to better outcomes and greater satisfaction among patients and employees, as well as to lower costs of treatment and care (17). We found that leaders’ evaluation of quality corresponded neither with employees’ evaluation nor with hospital regulations. The employees thought that leaders did not have enough innovative suggestions to improve the quality of their work. Leaders should encourage employees’ involvement and include their knowledge in improving the quality of nursing (25).

Nursing leaders who participated in the study were aware that they did not have enough management and leadership knowledge. Most of them had not acquired knowledge before taking up a leadership position, which means that they either acquired it later or that they only improved it with workplace experience, which was also found by other studies (35,36). In our case, almost half of the nursing leaders still used an inappropriate leadership style in their work. Both nursing leaders and employees thought that there should be a definition of nursing managerial competencies. Also, teaching of these competencies should be included in Slovenian educational system along with teaching of professional skills.
