# Supplementary material for: Perceptions of managerial competencies, style, and characteristics among professionals in nursing
Source: Croat Med J. 2011 Apr;52(2):198–204. doi: 10.3325/cmj.2011.52.198 (PMC3081218; doi:10.3325/cmj.2011.52.198)
Supplement: Supplementary Table 1 [file CroatMedJ_52_s002.pdf]

QUESTIONNAIRE FOR LEADERS

.

Demografic data:	Year of employment:	Level of education :
Gender **M** **F**<br>Age ………….year.<br>Institution of work:<br>………………………...<br>Year of employment in this institution:……………year.<br>Year of leading position:<br>………….year .	1. <5 let<br>2. 5 - 10 let<br>3. 11 - 15 let<br>4. 16 - 20 let<br>5. 21- 25 let<br>6. 26 - 30 let<br>7. > 30let	1. SECONDARY SCOOL<br>2. HIGH SCHOOL<br>3. COLLEGE<br>4. UNIVERSITY LEVEL<br>5. MASTER LEVEL<br>6. PhD

**Each claim that describe you or your co-workers mark with suitable assessment**	**Totally disagree**	**Disagree**	**Partly agree**	**Agree**	**Totally agree**
1.I consistently work on standard procedures.	**1**	**2**	**3**	**4**	**5**
2.Co-workers respect me.	**1**	**2**	**3**	**4**	**5**
3.We are achiving asked goals on the department	**1**	**2**	**3**	**4**	**5**
4.I know how to recognize and identify problems.	**1**	**2**	**3**	**4**	**5**
5.In conflict situations I use a compromise.	**1**	**2**	**3**	**4**	**5**
6.I show personal interest for employees development.	**1**	**2**	**3**	**4**	**5**
7.It is necessary to avoid routine work	**1**	**2**	**3**	**4**	**5**
8.Introduction of changes is necessarily needed in hospital.	**1**	**2**	**3**	**4**	**5**
9.I encourage co-workers to improve the quality of work	**1**	**2**	**3**	**4**	**5**
10.Successful work requires mutual trust, support and communication.	**1**	**2**	**3**	**4**	**5**
11.Because improvements are not necessary, it is not necessary to promote initiatives	**1**	**2**	**3**	**4**	**5**
12.Working activities should be carefully organized	**1**	**2**	**3**	**4**	**5**
13.I allow that co-workers can show their abilities and knowledge.	**1**	**2**	**3**	**4**	**5**
14.Before I accept decision I introduce it to co-workers.	**1**	**2**	**3**	**4**	**5**
15.At decision making I use material rewards.	**1**	**2**	**3**	**4**	**5**
16.It is important to achieve good results regardless of the resources spent.	**1**	**2**	**3**	**4**	**5**
17.As a leader in nursing I have enough knowledge of leadership.	**1**	**2**	**3**	**4**	**5**
18.At solving conflict I consider my neutrality.	**1**	**2**	**3**	**4**	**5**
19.I always talk with co-workers about problems and solutions.	**1**	**2**	**3**	**4**	**5**
20.I informed co-workers about changes which effect on work	**1**	**2**	**3**	**4**	**5**
21.I consider the arrangements at work.	**1**	**2**	**3**	**4**	**5**
22.When something goes wrong, it is necessary to establish control.	**1**	**2**	**3**	**4**	**5**
23.Communication at department which I lead is spontaneous and running in all directions.	**1**	**2**	**3**	**4**	**5**
24.I am emphasing common interests and goals at solving conflicts.	**1**	**2**	**3**	**4**	**5**
25.I am solving problems continuously, that they aren't accumulating.	**1**	**2**	**3**	**4**	**5**
26.I take care that co-workers know the vision of our organization.	**1**	**2**	**3**	**4**	**5**

Each claim that describe you or your co-workers mark with suitable assessment	Totally disagree	Disagree	Partly agree	Agree	Totally agree
27.I reward collaborators adequately for work.	1	2	3	4	5
28.I ensure that employees are motivated.	1	2	3	4	5
29.I praise co-workers for the contributions of the team work.	1	2	3	4	5
30.When I delegate tasks to co-workers, I also trust them that they will finish tasks well.	1	2	3	4	5
31.I decided in accordance with my competencies and responsibilities.	1	2	3	4	5
32.I am always ready to help my superior.	1	2	3	4	5
33.I encourage initiative and innovation to improve quality.	1	2	3	4	5
34.I am giving to the partners feeling of equivalences at a conversation..	1	2	3	4	5
35.I am solving problems systematically.	1	2	3	4	5
36.At solving conflict I use power.	1	2	3	4	5
37.I offer co-workers possibility for professional development.	1	2	3	4	5
38.Good interpersonal relationships are important to me..	1	2	3	4	5
39.I encourage innovative proposals of my co-workers.	1	2	3	4	5
40.Betwen co-workers it is necessary to maintain friendly relationship.	1	2	3	4	5
41.I encourage team work for solving complex tasks.	1	2	3	4	5
42.I am confronting conflicts actively.	1	2	3	4	5
43.Material stimulations and rewards are important.	1	2	3	4	5
44.I always set realistic goals.	1	2	3	4	5
45.I equally consider all proposals to solve the problem.	1	2	3	4	5
46.I forward to the co-workers all necessary information in time.	1	2	3	4	5
47.I get a suitable payment for my work.	1	2	3	4	5
48.I am using different pressures at deciding (hazing, reproaches, transfer,…).	1	2	3	4	5
49.At the department, which I lead, we have good interpersonal relationships.	1	2	3	4	5
50.I delegate also more demanding tasks that require a higher level of responsibility.	1	2	3	4	5
51.Our organization has a good reputation in public.	1	2	3	4	5
52.I accept responsibility for may decisions.	1	2	3	4	5
53.I always praise good work.	1	2	3	4	5
54.When I introduce a new task, co-workers are willing to actively participate.	1	2	3	4	5
55.I know key values of our organization.	1	2	3	4	5
56.When the organization is successful, the introduction of changes is not necessary.	1	2	3	4	5
57.I gained knowledge of leadership per wish of employer.	1	2	3	4	5
58.Employees are the most important wealth that our hospital has.	1	2	3	4	5
59.I take care of pleasant working atmosphere.	1	2	3	4	5
60.Because solving conflict is uncomfortable, I try to avoid them.	1	2	3	4	5
61.I encourage members of team that they help and support themselves.	1	2	3	4	5
62.I assigned tasks to co-workers that they are challenge for them.	1	2	3	4	5
63.I tell my views and co-workers must follow them.	1	2	3	4	5
64.At decision making I use my personal power.	1	2	3	4	5
65.When I introduce changes I predict potential obstacles	1	2	3	4	5
66.I am talking with co-workers a lot, through conversation I know them better.	1	2	3	4	5
67.I encourage co-workers to suggest possible solutions of the problem	1	2	3	4	5
68.Co-workers on the department can tell what they think.	1	2	3	4	5
69.At solving conflicts I am looking for a solution with co-workers.	1	2	3	4	5
70.Co-workers knows key values of our organization	1	2	3	4	5
71.I assign working tasks to co-workers according to their knowledge.	1	2	3	4	5
72.At decision making I use nonmaterial rewards.	1	2	3	4	5
73.My messages to co-workers are clear and understandable.	1	2	3	4	5
74.Co-workers can use their knowledge, abilities and skills.	1	2	3	4	5
75.I express anger an inappropriate manner	1	2	3	4	5
76.I give opportunity to co-workers to participate in the working team.	1	2	3	4	5
77.The leadership knowledge I have gained before I take a leadership position	1	2	3	4	5
78.I am proud that I work in this organization.	1	2	3	4	5
79.I encourage innovative proposals of co-workers.	1	2	3	4	5
80.I praise co-workers, because it effects on their satisfaction.	1	2	3	4	5
81.My leadership is improved since then I became a leader.	1	2	3	4	5
82.I accept professionally established decisions.	1	2	3	4	5

Each claim that describe you or your co-workers mark with suitable assessment	Totally disagree	Dusagree	Partly agree	Agree	Totally agree
83.When I scold, it is clear that something was made wrong.	1	2	3	4	5
84.I am consistent at complying rules.	1	2	3	4	5
85.I think that my working place is respectable.	1	2	3	4	5
86.I adapt my communications to different people and situations.	1	2	3	4	5
87.I use the power management hierarchy at decision making.	1	2	3	4	5
88.It is necessary to considered superior.	1	2	3	4	5
89.I listen and I consider suggestions of co-workers.	1	2	3	4	5
90.My way of communication have a positive impact on interpersonal relationships.	1	2	3	4	5
91.I wouldn't leave organisation, although I would get better job.	1	2	3	4	5
92.Employees in nursing must continually educate.	1	2	3	4	5
93. Leadership is responsible and accountable for making changes.	1	2	3	4	5
94. I represent a vision of our organization with my behavior	1	2	3	4	5
95.I argue my decisions suitable.	1	2	3	4	5
96.Agreement is base of mutual communication.	1	2	3	4	5
97.I recognize experiencing emotions of other people.	1	2	3	4	5
98.I am always willing to help my co-workers.	1	2	3	4	5
99.Every leader in nursing need additional knowledge of leadership.	1	2	3	4	5
100.Co-workers trust me.	1	2	3	4	5
101.Praise of good work I present in public.	1	2	3	4	5
102.Changes are easiest in knowledge, heavier in values and most difficult in knowledge.	1	2	3	4	5
103.My development in the hospital is in line with my personal goals.	1	2	3	4	5
104.In emotionally straining and stressful situations I keep sensibleness.	1	2	3	4	5
105.I encouraged co-workers to participate in working tasks.	1	2	3	4	5
106.Employees supporting each other and are prepared to help.	1	2	3	4	5
107.I encourage members of team, that they work for common goals.	1	2	3	4	5
108.At introducing changes I offer appropriate support to co-workers.	1	2	3	4	5
109.I speak only positively about our organization	1	2	3	4	5
110.I am ruled for consistent derivation of tasks and goals.	1	2	3	4	5
111.I am open for constructive criticism.	1	2	3	4	5
112.Tecnics and technology are essential for success.	1	2	3	4	5
113.Co-workers are independence about delegated tasks.	1	2	3	4	5
114.I believe in the successful development of our organization.	1	2	3	4	5
115.I give criticism to everybody, but only when we are alone.	1	2	3	4	5
116.I use may professional knowledge at decision making.	1	2	3	4	5
117.Our hospital builds its growth on individual developing.	1	2	3	4	5
118.I make personal expression and personal development for co-workers.	1	2	3	4	5
119.I listen co-workers carefully.	1	2	3	4	5
120.Friendly relationships is not necessary essential for good working results.	1	2	3	4	5
121.I approach active at solving problems.	1	2	3	4	5
122.I am kind and indulgent to co-workers.	1	2	3	4	5
123.For successful leadership in nursing is necessary to define managerial competences.	1	2	3	4	5
124.Working with co-workers is closely linked to the strategy of our hospital.	1	2	3	4	5
125.I do what I am saying-.	1	2	3	4	5
126.I am imposing my opinion to the co-workers.	1	2	3	4	5
127.Making changes is always difficult for employees.	1	2	3	4	5
128.Making changes should be conducted only if the organization is in crisis.	1	2	3	4	5
129.I include co-workers in decisions about changes.	1	2	3	4	5
130.My work is useful and important.	1	2	3	4	5
131.I feel responsible for the results of main work.	1	2	3	4	5
132.My work effects at work of co-workers.	1	2	3	4	5
133.I am independent and autonomous at work.	1	2	3	4	5
134.I know the results of my work.	1	2	3	4	5

PERSONALITY CHARACTERISTICS	Totally disagree	Disagree	Partly agree	Agree	Totally agree		RANK (1-10)
**Decisiveness**	1	2	3	4	5		
**Initiative**	1	2	3	4	5		
**Innovation**	1	2	3	4	5		
**Ambition**	1	2	3	4	5		
**Persistence**	1	2	3	4	5		
**Communication**	1	2	3	4	5		
**Self-confidence**	1	2	3	4	5		
**Adaptability**	1	2	3	4	5		
**Activity at work**	1	2	3	4	5		
**Tact**	1	2	3	4	5		
**Thoughtfulness**	1	2	3	4	5		
**Honesty**	1	2	3	4	5		
**Sociability**	1	2	3	4	5		
**Reliability**	1	2	3	4	5		
**Objectivity**	1	2	3	4	5		
**Cooperation**	1	2	3	4	5		
**Teamwork**	1	2	3	4	5		
**Organization skills**	1	2	3	4	5		
**Responsibility**	1	2	3	4	5		
**Emotional intelligence**	1	2	3	4	5		
